# Hyponatraemia and cirrhosis

**DOI:** 10.1093/gastro/got037

**Published:** 2014-01-21

**Authors:** Robert J. Gianotti, Andres Cardenas

**Affiliations:** ^1^Department of Gastroenterology, Beth Israel Deaconess Medical Center, Boston MA and ^2^GI Unit, Institut Clinic de Malalties Digestives i Metaboliques, Hospital Clinic, University of Barcelona.

**Keywords:** hyponatraemia, cirrhosis, end-stage liver disease, vaptans

## Abstract

Hyponatraemia is a common complication of advanced cirrhosis related to an impairment in the renal capacity for eliminating solute-free water, causing a retention of water that is disproportionate to the retention of sodium, thus leading to a reduction in serum sodium concentration and hypo-osmolality. The main pathogenic factor responsible for hyponatraemia is a non-osmotic hypersecretion of arginine vasopressin (AVP) or antidiuretic hormone from the neurohypophysis, related to circulatory dysfunction. Hyponatraemia in cirrhosis is associated with increased morbidity and mortality. Hyponatraemia is also associated with increased morbidity and impaired short-term survival after transplantation. The current standard of care based on restricting fluids to 1–1.5 L/day is rarely effective. Other approaches, such as albumin infusion and the use of vaptans—which act by specifically antagonizing the effects of AVP on the V2 receptors located in the kidney tubules—have been evaluated for their role in the management of hyponatraemia. The short-term treatment with vaptans is associated with a marked increase in renal solute-free water excretion and improvement of hyponatraemia; however their use in patients with end-stage liver disease is limited by hepatotoxic effects of some of these drugs. Long-term administration of vaptans seems to be effective in maintaining the improvement of serum sodium concentration, but the available information is still limited.

## INTRODUCTION

Hyponatraemia is frequently encountered in patients with end-stage liver disease. The incidence and severity are variable, but have been shown to occur in up to 57% of hospitalized patients with cirrhosis [1]. An understanding of the pathophysiology of low serum sodium in patients with end-stage liver disease is integral to management in both the inpatient and outpatient settings. Hyponatraemia has been shown to be an independent predictor of poor outcomes in cirrhosis and is often associated with refractory ascites, spontaneous bacterial peritonitis, and hepatic encephalopathy [1–3].

## PATHOPHYSIOLOGY

Hyponatraemia is defined as a serum sodium level ≤136 mEq/L [4] while, in cirrhosis, it has classically been considered relevant only at a serum sodium level <130 mEq/L [5]. In general, hyponatraemia can be divided into three clinical types: hypovolemic, euvolemic and hypervolemic, with some patients presenting a mixed picture. In this review, we will focus on the case of hyponatraemia that occurs in the setting of a hypotonic serum and increased extracellular fluid volume, or so-called ‘dilutional hyponatraemia’. In this instance, there is a distinct impairment of free water excretion in the presence of excessive anti-diuretic hormone (ADH). Diseases associated with this of this type of hyponatraemia include—but are not limited to—cirrhosis, congestive heart failure, certain types of renal failure, and nephritic syndrome [4].

Hyponatraemia in cirrhosis largely occurs in the setting of expanded extracellular fluid volume. There are of course important instances where a patient will present with hypovolemic hyponatraemia in the setting of diuretic use or gastrointestinal losses. When evaluating a cirrhotic patient with low serum sodium, it is important to exclude and treat these causes as the sole or major contributing factor.

In cirrhosis, total body water stores are increased, yet effective arterial volume is decreased [6]. The decrease in effective arterial volume is a product of splanchnic arterial vasodilatation that is mediated by excessive production of nitric oxide and other vasodilator compounds, such as endotoxin, substance P, and endogenous cannabinoids, in the setting of increased intrahepatic resistance [7, 8]. This process leads to sodium avidity in the proximal portion of the nephron, by activation of the renin–angiotensin–aldosterone axis and excess ADH-mediated free water re-absorption in the collecting tubule. Arterial side baroreceptors, found in areas such as the left ventricle and the carotid sinus, have been shown to be a potent regulator of ADH secretion that can overcome the suppressive effects of hypo-osmolality [6]. In the cirrhotic patient with ascites, the non-osmotic release of ADH from the posterior pituitary becomes the dominant force and the end result is impaired free water excretion and subsequent dilutional hyponatraemia.

The action of ADH on the kidney occurs predominantly in the principle cells of the collecting tubule. The stimulation of the vasopressin receptor, V2, by ADH leads to the downstream activation of a cyclic AMP-based pathway and subsequent up-regulation of the aquaporin channel, AQP2, in the apical membrane of the principle cell [9]. This allows the free flow of water from the tubular fluid back into the circulation [10].

## CLINICAL SIGNIFICANCE

Hyponatraemia in cirrhosis has been clearly described as an independent risk factor for mortality and is common in patients with end-stage liver disease [3, 11]. A 2006 study of 997 cirrhotic patients demonstrated a prevalence of serum sodium level ≤130 mmol/L of 21.6% [1]. This patient subgroup had a significantly higher incidence of hepatic encephalopathy (OR = 3.40; 95% CI: 2.35–4.92), hepatorenal syndrome (OR = 3.45; 95% CI: 2.04–5.82), and spontaneous bacterial peritonitis (OR = 2.36; 95% CI: 1.41–3.93). There was also a higher rate of refractory ascites and requirement for frequent therapeutic paracentesis proportional to the level of serum sodium <135 mmol/L. Serum sodium and the Model for End-stage Liver Disease (MELD) score have both been shown to predict mortality in patients with advanced cirrhosis on the liver transplant waiting list [12, 13]. Combining serum sodium with MELD (MELD-Na) was shown to more accurately predict mortality on the waiting list, compared with MELD score alone. This was particularly true in patients with lower overall MELD scores.

Low serum sodium has been shown to have a negative impact on the quality of life in patients with cirrhosis and ascites. A recent cross-sectional study of 523 patients with cirrhosis complicated by ascites demonstrated that health-related quality of life (HRQL) was significantly decreased in patients with hyponatraemia and serum sodium less than 130 mEg/L [14]. This effect was independent of disease severity marked by liver failure or increased MELD score. Interestingly, there was a significant impairment in the HRQL, even in patients with mild hyponatraemia, with serum sodium falling between 130 mEq/L and 135 mEq/L. In addition, recent data point to hyponatraemia as a strong predictor of poor HRQL, independent of overt cognitive dysfunction, and this may be improved following withdrawal of diuretics in the sub-group of patients whose serum sodium responds to this intervention [2].

Hepatic encephalopathy has been shown in several studies to be worsened by the presence of hyponatraemia. Hyponatraemia with serum sodium ≤130 mEq/L is one of several predictive factors, along with a history of encephalopathy, serum creatinine and bilirubin, for the development of overt hepatic encephalopathy in a one year study period [15]. Hyponatraemia was the strongest predictive factor with an HR of 10.5 (95% CI: 5.4–20.3) for development of overt encephalopathy in one year, and was also found to be correlated with decreased levels of the brain osmolyte, myo-inositol, supporting a role for cerebral intracellular water shifts and astrocyte swelling in the development of hepatic encephalopathy. Overt encephalopathy occurring after trans-jugular portosystemic shunt (TIPS) placement for variceal bleeding or refractory ascites has also been shown to occur with greater frequency in those patients entering the procedure with a serum sodium level ≤135 mEq/L [16].

The brain is uniquely adapted to counteract the effects of hyponatraemia. In situations of low extracellular osmolality, water will flow from the extracellular compartment down the osmotic gradient into astrocytes, causing oedema. In addition to the outward flow of cations, such as potassium, there are a number of organic compounds, including myo-inositol, that can counter this effect by being transported out of the intracellular space to equilibrate the osmolality between compartments [17]. Myo-inositol is also transported out of astrocytes to oppose the accumulation of glutamine that occurs in the setting of hyperammonemia in cirrhosis. This depleted state may predispose patients to worsening hepatic encephalopathy and astrocyte swelling with oxidative stress in the setting of hyponatraemia [18]. In fact, it has been demonstrated that cirrhotic patients with hypo-osmolarity—with and without hepatic encephalopathy—have depleted cerebral levels of myo-inositol as demonstrated on 1H-MR spectroscopy [19].

Hyponatraemia is a marker of poor outcomes in patients hospitalized with infections. Spontaneous bacterial peritonitis (SBP) is often associated with significant morbidity, including renal failure, and has a high mortality rate in published series [20, 21]. Patients with hyponatraemia at diagnosis of SBP are at much higher risk for development of hepatorenal syndrome and death [20]. The incidence of hyponatraemia and renal failure in cirrhotic patients admitted for skin and soft tissue infection has also been shown to be higher than in matched cirrhotic controls without infection, and was associated with higher 3-month mortality, compared with patients who had not developed hyponatraemia and renal failure (45% vs 19%) [22].

Patients awaiting liver transplant with hyponatraemia have also been shown to have poorer outcomes when compared with normonatremic controls. Using data derived from the Organ Procurement and Transplantation Network, Kim *et al.* developed and validated a survival score that included serum sodium in the model for end-stage liver disease (MELD-Na) [12]. Serum sodium was found to independently predict mortality with an HR of 1.05 per mmol decrease in serum sodium between 125 mmol/L and 140 mmol/L. The combination of MELD and serum sodium was significantly higher than MELD score alone in 7% of patients who died within 3 months of being listed for transplantation. This result suggested that there was a subgroup of patients that would benefit from gaining sodium-based exception points that may expedite time to transplantation. Hyponatraemia was also recently shown to predict mortality in the first 90 days after listing for transplant in a paediatric population [23].

Pre-transplantation serum sodium level has been shown to predict overall poor outcomes and increased mortality following orthotopic liver transplant. In one series there was a significant increase in the relative number of cases of central pontine myelinolysis following transplant in those patients with serum sodium <125 mmol/L, compared with those with normal serum sodium level (4.6% vs 0.1%; *P < *0.01) with a low absolute number of cases among all patients receiving transplant (0.5%) [24]. One series of 241 patients listed for transplant evaluated the effect of hyponatraemia (Na <130 mEq/L) on early post-transplant outcomes including 3-month survival and complications including infection, neurological disease and renal failure [25]. Post-transplantion survival at 3 months was significantly reduced in those patients with hyponatraemia prior to transplant (84% vs 95%; *P < *0.05) with equivalent survival following this period. The probability of renal failure, neurological disease and infectious complications were also increased with OR 3.4, 4.6 and 2.7, respectively. A similar result was found in a large cohort of 5152 transplant recipients from the United Kingdom and Ireland [26]. In this series, patients undergoing transplant with hyponatraemia and a serum sodium <130 mEq/L were found to have a higher risk-adjusted mortality at 90 days post-transplant, compared with controls with normal sodium (HR = 1.55; 95% CI: 1.18–2.04).

## TREATMENT

Treatment options for patients admitted with hyponatraemia and cirrhosis are limited. The first step in management should be to identify and correct the underlying cause of hyponatraemia, which includes holding diuretics and addressing gastrointestinal losses. Patients who are found to be hypovolemic with correlate orthostasis or pre-renal azotemia should be adequately resuscitated with intravenous crystalloid or albumin infusion. It is important to recognize that the cirrhotic patient with true hypovolemic hyponatraemia without ascites or oedema is rare [5].

The level of serum sodium at which to institute treatment has not been defined in clinical trials. The benefit of treatment must be weighed against the potential risks of correcting serum sodium too rapidly and running the risk of the rare—albeit severe—complication of osmotic demyelination. Generally, patients with serum sodium <130 mEq/L should be considered for treatment.

Severe hyponatraemia with serum sodium level <120 mEq/L is uncommon in the cirrhotic population, occurring in less than 1.2% of patients [27]. In the setting of severe hyponatraemia with symptoms such as seizure, one must consider correction to a safe level, so as to prevent recurrence and neurological injury. This is the one situation where the administration of hypertonic saline is advised, with care taken to avoid overly rapid correction. Serum sodium increases of <10 mmol/L in 24 hours and < 18 mmol/L in 48 hours are recommended [28]. The use of hypertonic saline in cirrhotic patients can lead to worsening ascites and oedema secondary to the sodium avid state that exists in the nephron, and should be used only in acute situations.

Albumin infusion can be considered as a treatment for hyponatraemia in cirrhosis. The available data are limited and include a small number of patients with very short-term follow-up, but suggest a benefit that needs to be explored in larger, randomized trials [29, 30]. A randomized pilot study in 24 patients admitted with serum sodium <130 mmol/L found that albumin significantly improved serum sodium levels, compared with matched controls treated with fluid restriction, with a mean increase of 9 mmol/L. There was also a significant increase, compared with controls, in free water clearance and serum vasopressin levels in patients treated with albumin [29]. This finding suggests that albumin may contribute to the improvement of circulatory dysfunction and decrease the non-osmotic release of arginine vasopressin (AVP).

Free water restriction to less than 1.0–1.5 L/day has become standard practice in treating patients with hypervolemic hyponatraemia in cirrhosis and may have some anecdotal benefit in preventing a further drop in serum sodium [5]. Trials including fluid-restricted control groups (<1.5 L/day) have demonstrated no significant benefit in free water clearance or serum sodium [31, 32]. This method has not been proven in clinical trials, is often very difficult to monitor and many patients cannot realistically adhere to this restriction.

The introduction of a new drug class, termed ‘vaptans’, was heralded by excitement over their potential benefit in cirrhosis-related hyponatraemia. These medications act as direct antagonists of the V2 receptor in the collecting tubule of the nephron, and significantly increase free water clearance. Tolvaptan (Samsca) is currently the only orally administered V2R antagonist that is approved for use in the United States. Lixivaptan and sativaptan have also been studied in cirrhosis and hyponatraemia.

The efficacy of tolvaptan in raising serum sodium level was studied in two randomized, placebo controlled, double blind phase 3 trials (Study of Ascending Levels of Tolvaptan in Hyponatraemia 1 and 2 [SALT-1 and SALT-2]) [33]. All patients had dilutional hyponatraemia with serum sodium ≤135 mEq/L, with 50% of patients classified as markedly hyponatraemic with serum sodium levels <130 mEq/L. The population was not limited to patients with cirrhosis (22.4% SALT-1 and 30.5% SALT-2) but included those with hyponatraemia related to heart failure and the syndrome of inappropriate anti-diuretic hormone (SIADH). All patients were hospitalized and were randomized to tolvaptan 15 mg daily or placebo, with up-titration of dosing to a maximum of 60 mg/d in those who failed to respond to lower doses. Serum sodium improved and reached normal levels in a significantly larger number of patients in the tolvaptan group, compared with placebo (*P < *0.001). The most common side-effects were thirst and dry mouth. An important subgroup analysis of the patients with cirrhosis revealed a significant increase in free water clearance, associated with weight loss, without renal impairment and normalization of serum sodium to >135 mEq/L in 41% of patients at day 4 and 33% at day 30 [34]. A secondary analysis also found a significant improvement in health-related quality of life scores in patients treated with tolvaptan. Hyponatraemia reliably recurs upon discontinuation of treatment. That said, recent concerns over the safety of tolvaptan were raised by a subanalysis of a large, multicentre trial that evaluated the use of tolvapatan in patients with polycystic kidney disease [35]. Patients developed a significant elevation of liver enzymes; thus the FDA placed a ‘black box’ warning on the drug, limiting its use on patients with liver disease.

Satavaptan and lixivaptan have been evaluated in several trials of hyponatraemia, including in cirrhotic patients, who showed an improvement in hyponatraemia and no serious adverse events compared with placebo [31, 32, 36]. Long-term data on the use of vaptans in cirrhosis is limited but, in trials of SIADH, both satavaptan and tolvaptan had lasting effects and maintained near-normonatremia when continued for one year [37]. Satavaptan was shown to maintain efficacy throughout a one-year study period in a cohort of 73 cirrhotic patients treated with drug vs placebo, with no significant increase in adverse events [38]. Unfortunately, the use of satavaptan was associated with an increased mortality in one of the studies—though not in the other two—and the drug was withdrawn from development. The reason for this increased mortality could not be elucidated. It is not known if this increased mortality during long-term treatment is a class effect or exclusively related to satavaptan.

Overall, the vaptans have not been shown to improve outcomes in cirrhosis. A recent meta-analysis evaluated outcomes in 2266 patients from 12 randomized trials of tolvaptan, satavaptan and lixivaptan. The primary outcome measure was mortality and secondary outcomes included—but were not limited to—complications of cirrhosis and mobilization of ascites [39]. While the vaptans did increase serum sodium levels during all study periods and improved ascites mobilization as exemplified by reduced mean body weight (mean difference of −1.82 kg) and increased time to first large volume paracentesis (RR = 0.76; 95% CI: 0.60–0.83), there was no mortality benefit (RR = 1.06; 95% CI: 0.90–1.26). There was a significant increase in thirst (RR = 3.97; 95% CI: 1.78–8.83) and excessive urine volume of >5 L/day (RR = 9.96; 95% CI: 1.38–71.68). These adverse effects are important, particularly in a patient population that is predisposed to hepatic encephalopathy, limiting access to water and physical deconditioning limiting mobility. Based on the lack of clear benefit and lingering questions of risk, the routine use of vaptans in cirrhosis is not recommended.

The effects of vaptans on control of ascites and prevention of hepatic encephalopathy has been evaluated in several trials. In a study of 1200 patients combined from three randomized trials with (a) uncomplicated ascites, (b) difficult-to-treat ascites with- and (c) without diuretics, there was no significant benefit from satavaptan in preventing worsening of ascites or reducing the number of large volume paracentesis. Satavaptan did improve serum sodium level when compared with placebo and also showed a small but significant increase in time to first large-volume paracentesis (RR = 0.72; 95% CI: 0.53–0.98) [40]. A meta-analysis of this same patient population failed to show a significant decrease in the number of hepatic encephalopathy episodes with satavaptan [41]. A recent trial in Japan randomized 164 patients to receive either tolvaptan or placebo as add-on therapy to diuretics, with the primary end-point of weight change at 7 days. There was a significant reduction in weight in the tolvaptan group, compared with placebo (-1.95 kg vs -0.44 kg; *P < *0.0001), and this benefit held true, even in patients with serum albumin <2.5 g/dL [42].

## CONCLUSION

Hyponatraemia is a commonly encountered problem in patients with end-stage liver disease. Low serum sodium is a poor prognostic indicator in both the pre- and post-transplant patient population and has been shown to increase the risk of early mortality and complications including infection, renal failure, and encephalopathy. The treatment options for hyponatraemia are limited and are currently based on adequate free water restriction, cessation of diuretics, and potentially the use of vaptans in the short term on a patient-by-patient basis. The vaptans may be of potential benefit in the peri-transplant period; however, drugs currently on the market should not be used for this indication in patients with cirrhosis. This issue therefore needs to be further evaluated with new vaptan agents in controlled trials. Liver transplantation remains the only definitive treatment for end-stage liver disease complicated by hyponatraemia. A recommended algorithm for the management of hyponatraemia in patients with cirrhosis is depicted in Figure 2.

**Figure 1.**
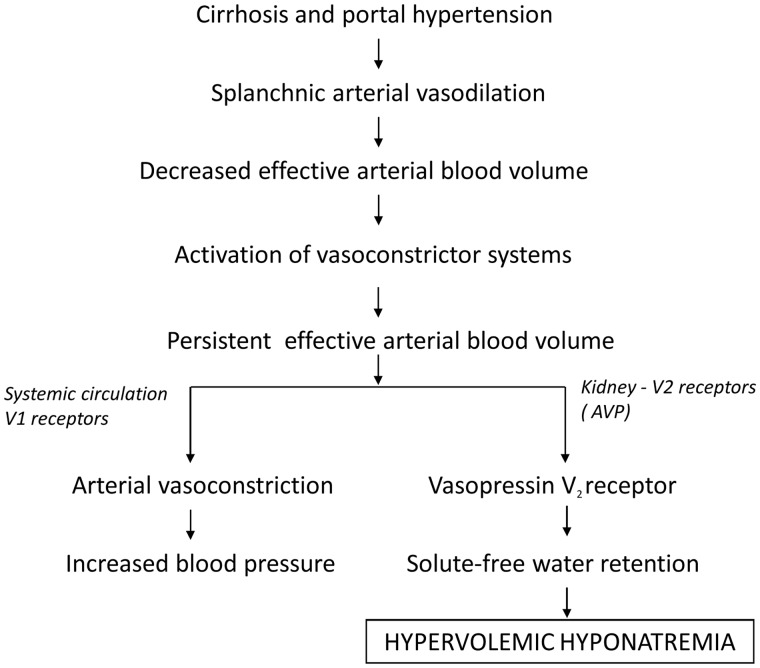
Proposed pathogenesis of hypervolemic hyponatraemia in cirrhosis. There is activation of the renin—angiotensin—aldosterone system (RAAS) and sympathetic nervous system (SNS) and a non-osmotic hypersecretion of arginine vasopressin (AVP) due to decreased effective arterial blood volume that activates baroreceptors and stimulates the hypothalamic release of AVP, causing renal solute-free water retention through the action of V2 receptors and arterial vasoconstriction through the action of V1 receptors.

**Figure 2.**
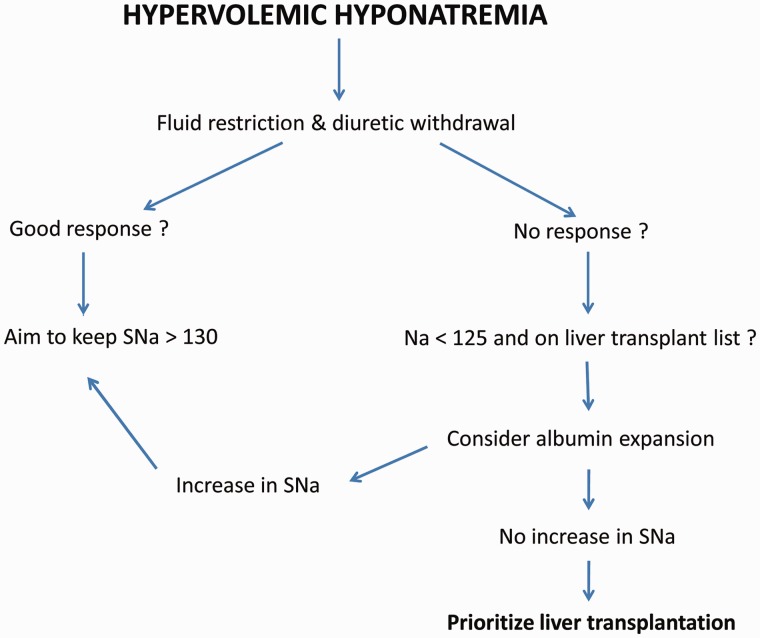
Proposed algorithm for the management of hyponatraemia in cirrhosis. Fluid restriction up to 1–1.5 L/day if hyponatraemia persists despite diuretic withdrawal. Albumin at a dose of 40 mg/day for 7–14 days.

**Conflict of interest:** none declared.
